# Measures for disinfection and sterilization as the most significant concept in health care infection control system in Russia

**DOI:** 10.1186/2047-2994-4-S1-P47

**Published:** 2015-06-16

**Authors:** N Shestopalov, V Akimkin, L Panteleeva, L Fedorova, I Abramova

**Affiliations:** 1 Federal Budget Scientific Institution “Scientific Research Disinfectology Institute” of Federal Service for Surveillance on Consumer Rights Protection and Human Well-being, Moscow, Russian Federation; 2 State Budget Educational Institution of Higher Professional Education “I.M. Sechenov’s First Moscow State Medical University”, Moscow, Russian Federation

## Introduction

Under current conditions of development of health care and generally mankind, prevention of healthcare-associated infections (HAI) is one of the global world problems.

## Objectives

Prophylaxis of HAI in Russia.

## Methods

Epidemiological, statistical.

## Results

HAI affect 5-10% of patients staying at in-patient departments and rank the tenth place among the causes of population mortality. Annually according to the data of official statistics in Russia approximately 30,000 of HAI cases are registered but the experts consider their actual quantity to be not less than 2-2,5 mln of people.

Under current conditions in Russia “National Concept of Prevention of Health Care Associated Infections” (2011) was enacted and is valid. The present concept was developed by specialists of Federal Service for Surveillance on Consumer Rights Protection and Human Well-being, famous scientists and health professionals, and it determines the purpose, principles, general architecture, major improvement concepts of national system of health care associated infections prevention, its functioning mechanisms and also expected social-and-economic effect.

## Conclusion

Taking into account absence of specific means and prevention issues solution methods one of the most important concepts of HAI control is improvement of disinfection and sterilization methods. This concept in Russia is particularly significant due to wide spread of microorganism with high resistance to the effect of majority of antibiotics and antiseptics groups, development of modern technologies (Impulsive Ultraviolet Light, Aerosol Disinfection, etc.), building and reconstruction of centralized sterilization in-patient departments with updated equipment for washing and disinfection of medical products and endoscopic devices.

## Disclosure of interest

None declared.

